# A comparative analysis of voluntary medical male circumcision before and during the COVID-19 pandemic in Gauteng province, South Africa

**DOI:** 10.4102/safp.v67i1.6062

**Published:** 2025-03-26

**Authors:** Cyril B. Fonka

**Affiliations:** 1 School of Public Health, Faculty of Health Sciences, University of the Witwatersrand, Johannesburg, South Africa

**Keywords:** COVID-19, voluntary medical male circumcision, HIV/AIDS, service disruption, Gauteng

## Abstract

**Background:**

Voluntary medical male circumcision (VMMC) is a significant biomedical and cost-effective intervention in preventing human immunodeficiency virus (HIV) transmission. This study aimed to compare VMMC before and during coronavirus disease 2019 (COVID-19) in Gauteng, one of the most affected provinces in South Africa by HIV and/or acquired immunity deficiency syndrome (AIDS) and COVID-19, to inform VMMC delivery and uptake during future pandemics.

**Method:**

A comparative analysis. Routine VMMC data were obtained from all public and private hospitals in Gauteng province from the District Health Information System and medical schemes. The datasets were merged with the years 2019 and 2020 as before and during COVID-19 periods, respectively. Percentage change in VMMC was calculated to determine changes in VMMC before and during the COVID-19 pandemic.

**Results:**

Provincially, VMMC declined in 2020 by an overall 33.8% for ≥ 10 years, 32.4% for 10–14 years and 35.8% for ≥ 15 years. All five districts in Gauteng province were affected differently. Exceptionally, the Tshwane Metropolitan district recorded an increase of 21.8% in ≥ 10 years and 36.0% in 10–14 years, despite a decline of –18.2% in ≥ 15 years in VMMC. While the other four districts saw a significant percentage decline in the three age groups of VMMC, the highest disruption was experienced in the West Rand district, in all three age groups.

**Conclusion:**

In 2020 during the COVID-19 pandemic, VMMC substantially declined in Gauteng province. However, Tshwane, one of the five districts in Gauteng experienced an increase in VMMC, highlighting a health system resilient lesson to be learned. The fight against HIV is crucial and warrants the continuum of VMMC during future crises.

**Contribution:**

The evidence may inform policies on VMMC delivery post-COVID-19 and particularly during future outbreaks as a strive to curb HIV transmission in South Africa.

## Introduction

South Africa is a sub-Saharan African country with the highest global prevalence of the human immunodeficiency virus and/or acquired immunity deficiency syndrome (HIV and/or AIDS), alongside the most extensive highly active antiretroviral therapy (HAART) programme worldwide, catering for over 8 million people living with HIV and/or AIDS.^1,2^ The benefits of the HAART include a reduction in HIV and/or AIDS mortality and the incidence of opportunistic infectious diseases^3^ and an improvement in life expectancy and quality of life (QoL) among people living with HIV and/or AIDS. Several interventions aimed at ending or at least halting HIV and/or AIDS incidence have included condom promotion, pre- and post-exposure prophylaxis (PrEP and PEP) and behaviour change like fidelity and abstinence and voluntary medical male circumcision (VMMC), among a combination of HIV prevention interventions.^4,5^

Voluntary medical male circumcision: the removal of the foreskin is a biomedical intervention with a 60% effectiveness in reducing the risk of HIV and/or AIDS transmission according to the World Health Organization (WHO) and the Joint United Nations Programme on HIV/AIDS (UNAIDS) based on evidence from a Ugandan Randomized Control Trial (RCT).^4,5^ This endorsement attracted invaluable support for VMMC in Africa from the United States (US) President’s Emergency Plan for AIDS Relief (PEPFAR).^6^ Additionally, VMMC is cost-effective in reducing the risk of HIV and/or AIDS acquisition across many sub-Saharan African countries including South Africa, with cost-saving and health benefits of infections and disability-adjusted life-years (DAYLs) adverted.^7,8^ Furthermore, VMMC is a once-off lifetime intervention compared to other HIV and/or AIDS interventions like daily antiretroviral medications.^9^ Likewise, VMMC also reduces the risk of sexually transmitted infections (STIs) like human papillomavirus (HPV), syphilis, Trichomonas and bacterial vaginosis and Mycoplasma genitalium infections among HIV and/or AIDS at-risk populations such as sex workers, men who have sex with men (MSM)-homosexuals and heterosexuals.^10^

Preponderance studies indicate that globally, the recent coronavirus disease 2019 (COVID-19) pandemic like previous outbreaks including the West African Ebola significantly disrupted multiple routine healthcare services,^11,12,13,14^ including VMMC across several sub-Saharan African countries with a potential negative consequence against HIV prevention.^15,16,17,18^ Coronavirus disease 2019 disruption of VMMC may have far-reaching effects such as an increased incidence of HIV transmission, which will require additional costs to treat.^15,17,18^ This phenomenon of essential service disruption is common in low- and middle-income countries (LMICs) with strained health systems because of resource constraints, often warranting the displacement of resources from essential services like HIV and/or AIDS, tuberculosis (TB) and malaria treatment and immunisation, among others, to tackle epidemics.^19^

Although Gauteng was one of South Africa’s provinces most affected by the COVID-19 pandemic^20^ and equally a province with a remarkable share of the country’s HIV and/or AIDS burden,^21^ several studies have examined the negative impact of the COVID-19 lockdown on other routine health services like maternal and child health and contraception distribution in the province,^14,22^ except for VMMC despite its significant efficacy in preventing HIV transmission. Research to fill this literature gap is of paramount importance and urgency to inform policy strategies for the continuum of VMMC during future outbreaks if the progress against HIV prevention is to be sustained. Moreover, such a study must highlight the geographical variation and the most affected area for the reallocation of resources and recovery support to restore VMMC uptake even above the pre-pandemic levels. Therefore, this study seeks to assess the indirect effect of COVID-19 on VMMC in Gauteng province by comparing VMMC before (2019) and during the COVID-19 pandemic (2020).

## Research methods and design

### Study design

This is a descriptive cross-sectional study, which allows the comparison of the number or percentage of VMMC before and during COVID-19 in Gauteng province and its districts. The term ‘post-COVID-19’ was avoided because the study period includes the year 2020, in which the pandemic was an existing major public health threat; hence the term or phrase before/pre- and during COVID-19 is preferred.

### Setting

The study was conducted in the Gauteng province of South Africa. Again, South Africa has the highest prevalence of HIV and/or AIDS worldwide with over 8 million people living with HIV and/or AIDS with a relative concentration in the Gauteng province although other provinces like KwaZulu-Natal lead in HIV prevalence.^1,2^ Gauteng province has a population of over 16 million and is the smallest but the most populated province in South Africa,^2^ attracting diverse groups of people from other parts of the country and externally, particularly from the Southern Africa Development Community (SADC), who are sexually active and at high risk of HIV contraction and transmission. This includes sex workers and MSM. Also, Gauteng province was one of the most affected provinces in South Africa by COVID-19, with the highest incidence and third highest mortality rates in the country,^20^ resulting in the disruption of routine services including HIV and/or AIDS treatment, maternal and child healthcare and contraceptive distribution to focus attention on COVID-19 patients.^14,22^ Gauteng province consists of five districts namely: The Johannesburg metropolitan municipality, which is the economic capital of the country, the Ekurhuleni metropolitan municipality, which houses the OR Tambo International Airport, the gateway into SADC, Tshwane Metropolitan municipality, which is the administrative headquarters of South Africa, and Sedibeng and West Rand districts. These health districts are supervised by the Gauteng Department of Health (GDoH). The diversity of Gauteng awards the province a unique character of generalisation of its studies across other provinces.

### Study population and sample

This study accounted for all VMMCs aged ≥ 10 years that were performed in all public and private hospitals in Gauteng province from 01 January 2019 to 31 December 2020. Voluntary medical male circumcision is subdivided into three groups: ≥ 10, 10–14 and ≥ 15 years. Equal or greater than 10 years is the total number of VMMC performed for males aged 10 years and above. While 10–14 years is VMMC performed for males strictly between the ages of 10–14 years. This follows for ≥ 15 years. Therefore, the 10–14 and ≥ 15 years are sub-groups in the total (≥ 10 years) number of VMMC. The categorisation of VMMC into ≥ 10, 10–14 and ≥ 15 years is a standard practice in South Africa. Because this was a population-level study that included all hospitals in the province, there was no need for a sample size calculation.

### Data collection

This study used longitudinal data collected from all public and private hospitals within Gauteng province from 01 January 2019 to 31 December 2020. Public hospital data are captured at individual healthcare facilities and inputted into an electronic database called the District Health Information System (DHIS), aggregations at the district and provincial level by the GDoH. The DHIS is used for monitoring and evaluation of health conditions and outcomes such as fully immunised, antenatal care (ANC), HIV testing, adherence and viral load suppression. Similarly, the private hospital data were captured at the hospitals and reported in the claims made to respective medical schemes, which in turn report to the Council of Medical Schemes as gatekeepers of the data. The author applied for the data from both sectors and it was provided in Microsoft (MS) Excel 2016 for 2019 and 2020. The data from both sources was then merged and cleaned with missing data checked before analysis. The data are stored in an encrypted Google Drive accessible to the researcher only.

### Analysis

Initially, the data were summed for the three age groups ≥ 10, 10–14 and ≥ 15 years for the years 2019 and 2020 as before and during COVID-19, respectively. To understand the real change or difference in VMMC before and during the pandemic, the formula (Equation 1) was used to calculate the percentage change in VMMC between the two periods and for the respective categories at the provincial and district levels. This formula or method has shown reliability in previous and similar studies that examined the percentage change in VMMC among 15 sub-Saharan African countries as a result of COVID-19 mitigation measures.^15^ Bar graphs were drawn in MS Excel to aid the visualisation and comparison of VMMC over the two study periods:

$$\begin{array}{l} \%\;chanage\;in\;VMMC=\\ \frac{Total\;number\;of\;VMMC\;in\;2020-Total\;number\;of\;VMMC\;in\;2019}{Total\;number\;of\;VMMC\;in\;2019}\\ x\frac{100}{1} \end{array}$$ [Eqn 1]

## Results

The results have been presented in two sections, the provincial and district levels.

### Provincial-level comparison of voluntary medical male circumcision before and during coronavirus disease 2019

Table 1 shows the longitudinal data of VMMC in Gauteng province from both the public and private hospitals over 2 years from 01 January 2019 to 31 December 2020. The difference between the two study periods is presented in the percentage (%) change column.

**TABLE 1:** Public and private sector voluntary medical male circumcision in Gauteng province.

VMMC category	Before COVID-19 (2019)			During COVID-19 (2020)			Change (%)
	Public hospitals	Private hospitals	Total	Public hospitals	Private hospitals	Total	
10–14 years	48 008	2547	50 555	33 385	797	34 182	−32.4
≥ 15 years	30 445	3028	33 473	19 262	2213	21 475	−35.8
≥ 10 years (total)	78 453	5575	84 028	52 647	3010	55 657	−33.8

VMMC, voluntary medical male circumcision.

Between 2019 and 2020, a total of 139 685 (84 028 + 55 657 = 139 685) VMMC (≥ 10 years) were performed in Gauteng province (Table 1). Of which the majority constituted 93.9% or 131 100 (78 453 + 52 647 = 131 100) were performed in public hospitals. While only 6.2% or 8585 (5575 + 3010 = 8585) were performed in medical aid accredited (private) hospitals.

**FIGURE 1:**
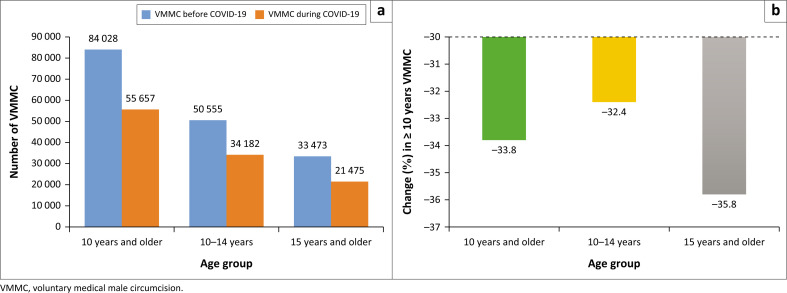
(a) Voluntary medical male circumcision in Gauteng province 2019–2020 and (b) change (%) in voluntary medical male circumcision in Gauteng province (2020).

Figure 1a shows a summary quantification of the decline in VMMC in Gauteng province during the COVID-19 pandemic in 2020 in all three aspects namely, the total number of VMMC ≥ 10, 10–14 and ≥ 15 years during COVID-19 periods, compared to the 2019 pre-COVID-19 era, from Table 1. In the baseline year 2019 before the COVID-19 pandemic, there was a total number (count) of 84 028 VMMC compared to a small amount of 55 657 VMMC during the COVID-19 period in 2020. Hence, a 33.8% decline (Figure 1b) in VMMC in Gauteng province in 2020 during the COVID-19 pandemic.

Also, all three age groups performed more VMMC before COVID-19 (in 2019) compared to the year of the pandemic in 2020. As such, VMMC in 10–14 years experienced a decline of 32.4% while ≥ 15 years saw a more noticeable decline of 35.8% during the COVID-19 pandemic (2020) as shown in Figure 1a. The highest decline (35.8%) was observed among the ≥ 15 years age group compared to the overall VMMC (≥ 10 years) performed in the province as well as in the 10–14-year age group.

Furthermore, it was interesting to investigate the effect of the pandemic on VMMC among the five districts in Gauteng province.

### District-level comparison of voluntary medical male circumcision before and during coronavirus disease 2019

Similarly, Table 2 presents the district performance of VMMC before COVID-19 and during COVID-19 and the percentage change for the five districts in Gauteng province. For simplicity and a better understanding of COVID-19’s effect on VMMC at the district level, we will focus our attention on the percentage change in VMMC during COVID-19.

**TABLE 2:** Gauteng district variation of voluntary medical male circumcision disruption because of coronavirus disease 2019.

District	VMMC before COVID-19 (2019)			VMMC during COVID-19 (2020)			Change (%) in VMMC [2020–2019/2019]*100		
	Total (≥ 10 years)	10–14 years	≥ 15 years	Total (≥ 10 years)	10–14 years	≥ 15 years	Total (≥ 10 years)	10–14 years	≥ 15 years
Johannesburg Metro	31 346	17 535	13 811	20 101	8193	11 908	−35.9	−53.3	−13.8
Tshwane Metro	18 736	13 839	4897	22 829	18 824	4005	21.8	36.0	−18.2
Ekurhuleni Metro	19 273	10 108	9165	6425	3413	3012	−66.7	−66.2	−67.1
Sedibeng	7917	5044	2873	5185	3331	1854	−34.5	−34.0	−35.5
West Rand	6533	3940	2593	1002	393	609	−84.7	−90.0	−76.5
Unclassified	232	114	118	64	9	55	−72.4	−92.1	−53.4

VMMC, voluntary medical male circumcision.

Generally, VMMC disproportionately declined during the COVID-19 pandemic in four districts except the Tshwane Metropolitan district, which experienced a positive performance during the pandemic. The district variation in VMMC is better unpacked with respect to the three age groups.

**FIGURE 2:**
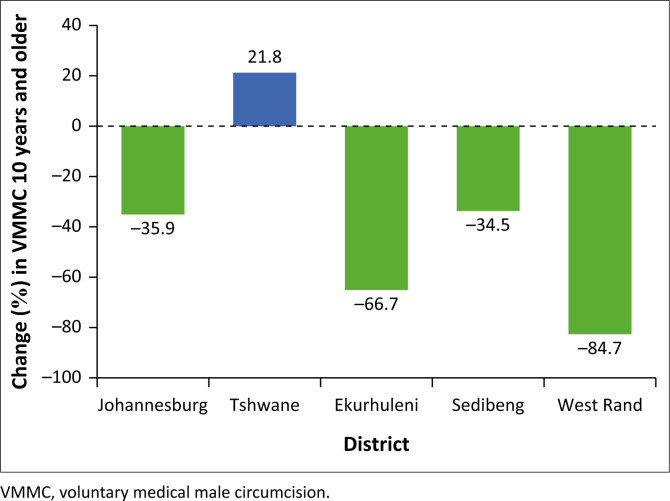
Gauteng COVID-19 district variation effect on voluntary medical male circumcision of ≥ 10 year olds.

Firstly, the overall percentage change in VMMC (≥ 10 years) during the COVID-19 pandemic in all five districts has been simplified and depicted in Figure 2. The Tshwane Metropolitan district had the only positive performance with an increase of 21.8% overall (≥ 10 years) during the COVID-19 period of 2020. In other words, in 2020 during the COVID-19 pandemic, the Tshwane Metropolis was the only district in Gauteng province that experienced a positive performance in VMMC compared to the other four districts. Of the four negatively impacted districts, the highest disruption of VMMC was observed in the West Rand districts, which amounted to an 84.7% decline. Followed sequentially by a decline of 66.7% in Ekurhuleni, 35.9% in Johannesburg and 34.5% in Sedibeng.

**FIGURE 3:**
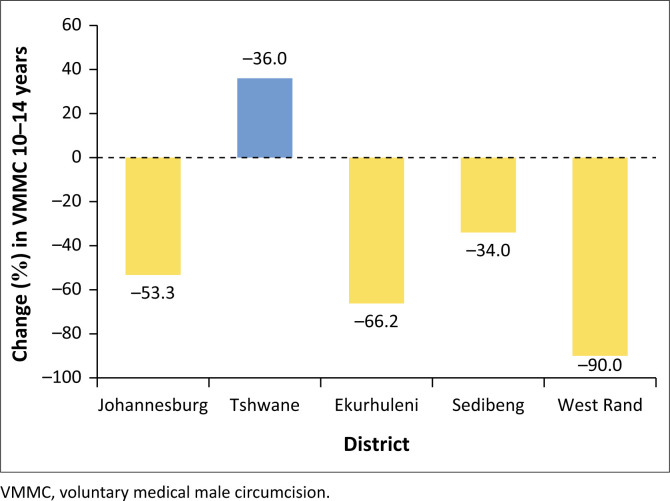
Gauteng COVID-19 district variation effect on voluntary medical male circumcision of 10-14 year olds.

Secondly, Figure 3 depicts the district variations in 10–14 years of VMMC. Again, the Tshwane Metro was the lone district that recorded an increase of 36.0% in 10–14-year VMMC in 2020 during the COVID-19 pandemic. Again, the previous sequence of decline observed in ≥ 10 years among the districts (West Rand, Ekurhuleni, Johannesburg and Sedibeng) repeats itself in 10–14 years.

**FIGURE 4:**
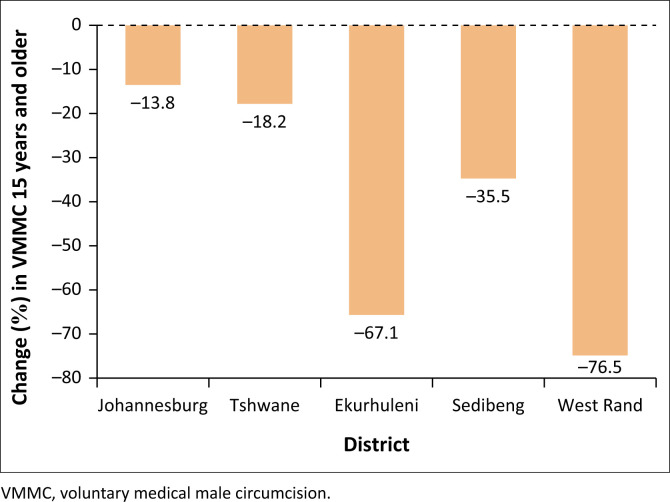
Gauteng COVID-19 district variation effect on voluntary medical male circumcision of ≥ 15 year olds.

Thirdly, Figure 4 shows the district variation in VMMC in ≥ 15 years in 2020 during the COVID-19 pandemic. Interestingly, all five districts experienced a decline in VMMC in ≥ 15 years during COVID-19 (2020) in the following chronological order; 76.5% in West Rand, 67.1% in Ekurhuleni, 35.5% in Sedibeng, 18.2% in Tshwane and 13.8% in Johannesburg. Remarkably, the Tshwane district that isolated itself with a positive performance in ≥ 10 years in Figure 2 and in 10–14 years in Figure 3 now experienced a decline in the ≥ 15-year age groups similar to the other four districts. Again, the West Rand district recorded the highest percentage decline in ≥ 15 years VMMC.

The unclassified VMMC in Table 2 with a corresponding decline in 2020 during COVID-19 represents data from the medical schemes that could not be classified in any of the districts because the postal code was unknown, but the province (Gauteng) was known. This adjustment is necessary as the district summation should equate to the provincial total. In both the provincial and district as well as the before and during COVID-19 analysis, the public sector decimally performed more VMMC than the private sector (medical scheme hospitals).

## Discussion

This study compared VMMC before (2019) and during the COVID-19 pandemic (2020) in Gauteng province and the district variations thereof. The results indicate that in 2020 during COVID-19, VMMC declined by 33.8% for ≥ 10 years, 32.4% for 10–14 years and 35.8% for ≥ 15 years, provincially. Four of the five districts in Gauteng experienced a decline in VMMC in all three age groups in 2020 during COVID-19. Exceptionally, the Tshwane Metropolitan district recorded an increase in VMMC of 21.8% overall (≥ 10 years) and 36.0% in 10–14 years compared to the pre-COVID-19 period of 2019. The highest decline in VMMC in all three age groups occurred in the West Rand district.

Overall, the current study observed a substantial decline in VMMC during the COVID-19 pandemic in 2020 in Gauteng province, compared to the pre-COVID-19 period of 2019. In the absence of any alternative explanation for the decline in VMMC during COVID-19 in the current study, and in appreciation of the multitude of studies that have correlated the mitigation strategies against COVID-19 like lockdowns hence movement restrictions, and the reallocation of health resources to the pandemic that adversely impacted a wide range of routine health services including HIV and/or AIDS, family planning, maternal and child healthcare and VMMC,^11,14,15,16,19^ the current study can attribute the decline in VMMC to the disruptive impact of the COVID-19 pandemic. For example, the 33.8% overall decline in VMMC in Gauteng province in 2020 during COVID-19 in the present study is consistent with a similar 31.8% decline in VMMC in another study that involved 13 Eastern and Southern African countries in 2020 compared to the baseline period of 2019.^16^ If COVID-19 could successfully disrupt elective surgery in a highly industrialised country with a robust health system like the US,^24^ everything being equal, it could have severely undermined VMMC in a resource-constrained Gauteng province and South Africa at large.^15^ Additionally, strong evidence from a mathematical model also highlighted the COVID-19 disruption of VMMC with both health and financial implications of additional HIV infections and treatment costs.^17^ Moreover, in 2020, PEPFAR discontinued support of VMMC for the 10–14 age group and significantly scaled back funding for VMMC programmes hence, the extent to which these changes affected VMMC service provision in Gauteng province, South Africa, and other LMICs should be examined by future studies. This shift of the PRPFAR support may be justified by the aligning evidence from a recent 2023 study that found VMMC to be more cost effective among 15–49-year-olds.^8^

The majority of the VMMC in the current study constituting 93.9% performed in public hospitals against 6.2% performed in private hospitals is indicative of socioeconomic dynamics such as affordability and accessibility. One interpretation is that in Gauteng province, which is indifferent to other low- and middle-income settings in sub-Saharan Africa, most VMMC clients prefer to access public healthcare facilities where the services are free of charge compared to private facilities where payment is required. On the other hand, this highlights the implication of accessibility, as private general practitioners are less available in rural settings; hence, rural communities often access health services like VMMC through public hospitals. Despite the large difference in the number of VMMCs performed between the public and private hospitals, both sectors need to work together to expand the geographical coverage of VMMC services to enhance the prevention of HIV transmission.

Also, the findings of the current study highlight mixed variations in VMMC during COVID-19 among the five districts in Gauteng province. Although a vast majority (four out of five) of the districts in Gauteng were adversely affected with the West Rand district experiencing the worst decline in all three age groups of VMMC in 2020 compared to the pre-COVID-19 period of 2019, its counterpart, the Tshwane district had an overall increase of 21.8% in ≥ 10 years because of a 36.0% increase in 10–14 years’ category. Such a mixed variation of COVID-19’s effect on VMMC corroborates with Peck et al.^15^ findings, wherein South Africa only achieved 30.7% of the annual target of VMMC in 2020, because of the COVID-19 pandemic compared to 2019. Whereas Rwanda recorded an additional surplus of 23% (123.0% national achievement) in VMMC during the same period-2020 to show the mixed effect whereby COVID-19 impacted VMMC differently in different settings.^15^ This could mean that unlike South Africa and the Gauteng province in particular, Rwanda may have better managed and enabled the delivery of VMMC during the pandemic cognisant of the undesired consequences of compromising HIV prevention. Interestingly, evidence suggests that COVID-19 disrupted essential maternal healthcare in Tshwane;^25^ however, VMMC in the district was spared as demonstrated by the current study. In the absence of literature to explain the exceptional increase in VMMC in Tshwane vis-à-vis the decline in other districts, the current study avoids speculations to this effect and rather recommends that further studies should explore the possible mechanism, reasons and leadership role that enabled the increased in VMMC in the Tshwane district during this critical period. This will assist other jurisdictions to benefit from the skillfulness of the Tshwane district in service delivery management to ensure the continuation of VMMC during future infectious disease outbreaks.

Therefore, the current study advocates for the scaling up of VMMC in Gauteng province and for stakeholder engagements to map out a framework for tackling the district variations. However, support for LMICs from institutions determined to end HIV and/or AIDS such as WHO, PEPFAR and the African Center for Disease Control (CDC), and local government through the Ministries of Health is critical for UNAIDS deadlines like the 2025 target of creating access to VMMC for 90% of eligible men and for ending HIV and/or AIDS by 2030, as well as the attainment of the sustainable development goal target 3.7 of enabling universal access to sexual and reproductive health for all by 2030.^26,27^ Even if these objectives are not attained at the designated timeframes, positive trends towards the VMMC targets will have healthier implications. Again, the current study emphasises a resounding call to improve the demand and supply of VMMC, which has a spillover effect to reduce the risk of horizontal transmission of HIV and/or AIDS. Efforts must continue in fighting vertical transmission of HIV and/or AIDS, including the Prevention of Mother-to-Child Transmission (PMTCT),^28^ to preserve and not undermine the pre-COVID-19 gains.

To improve VMMC, it is important to understand and address the factors hindering its accessibility besides movement restriction and the reallocation of health resources to mitigate the spread of COVID-19.^11,14^ This is critical because although COVID-19 has seized from being a public health threat, there is no guarantee that VMMC will automatically recover to the pre-COVID-19 trends. Evidence from Kenya suggests that the lack of policies and support for early infant circumcision, service integration at facilities, community ownership of VMMC programmes, domestic funding and household financial constraints undermine VMMC.^29^ Moreover, studies have highlighted counterperceptions acting as cognitive barriers to VMMC like perceived reduced sexual pleasure, sexual dysfunction, infertility and health workers’ misconceptions and negative attitudes among other factors.^30,31^ Although these claims have been refuted, such perceptions should be discussed during health promotion campaigns,^30,31^ coupled with addressing concerns about safety and adverse events.^9,32^

Therefore, to improve VMMC post-COVID-19 as well as to meet the UNAIDS target of scaling access to VMMC of 90% for all eligible men by 2025, it is worth wise to consider demand creation strategies to increase VMMC awareness and contemporary alternative methods of VMMC, for instance, the application of devices like situ for foreskin removal and elastic collar compression, contrary to surgery.^16^ Other VMMC demand creation strategies include, firstly, the scheduling of VMMC at male-dominated sectors like mines, farms, military residences and sports avenues.^9^ Secondly, the use of economic incentives like vouchers to compensate for transportation costs could improve VMMC.^9^ Thirdly, increase service delivery hours for VMMC and extend the services into communities, schools and higher education institutes.^9^ Fourthly, policy reviews should address barriers hindering access to VMMC for adolescent boys and men.^9^ In countries like South Africa and Malawi where traditional and cultural practices play a crucial role in circumcision,^30,33^ both clinical and traditional stakeholders should collaborate and integrate. Fifthly, better management of adverse events of VMMC could increase the number of young men willing to circumcise as reported in another South African study that involved the Gauteng province.^34^

Although the current study strictly focused on VMMC, the author argues that the fight against HIV and/or AIDS should be broader and more comprehensive, comprising other combination HIV prevention strategies like PrEP and PEP, particularly among at-risk groups such as MSM, sex workers and serodiscordant couples.^9,35^ Alongside VMMC, the efficacy and cost-effectiveness of condoms in preventing HIV and/or AIDS transmission in the South African context should be exploited.^36^ Furthermore, the 95-95-95 objective of HIV testing, treatment and viral load suppression should be incorporated into the VMMC package.^16,37^ Similarly, the integration of HIV services into mother and child healthcare will align with ‘The Global Alliance to End AIDS in Children by 2030’, by aiming to test, treat and care for HIV and/or AIDS cases among infants, children, pregnant women and breast-feeders and tackling gender, social and structural barriers in accessing healthcare will assist in ending HIV and/or AIDS.^28,37^ Lastly, community engagement and the inclusion of vulnerable populations like MSM and adolescent girls and women in the designing of interventions, sustainable funding for HIV and/or AIDS interventions and promulgating the right to care for the marginalised, discriminated, stigmatised and criminalised like MSM will advance ‘the end AIDS agenda’.^26,37^ Summarily, rescaling VMMC going forward is key to HIV prevention, but VMMC should not exist in isolation from other HIV prevention interventions.

### Study limitations and strengths

The main limitation of this study is that the dataset did not have potential confounders to control for other effects to fully justify the attribution of the decline in VMMC to the COVID-19 pandemic. Similar to all secondary data analysis and ecological studies, the quality of the data could not be improved. For example, we could not account for whether the decline in VMMC was because of speculations that a large proportion of men aged ≥ 10 years have already been circumcised within the province or whether the decline was an implication of the indirect effect of the COVID-19 pandemic. However, several previous studies have already established an inverse relationship between COVID-19 and routine service delivery globally, including VMMC.^11,13,14,15,16,17,18,19^ Also, there may have been a small-scale underreporting of VMMC during the study period, particularly for privately paying clients (not on medical schemes). Nonetheless, the impact that the missing data or underreporting could have on the current findings could be insignificant because the majority of VMMC is performed at public facilities, followed by medical aid accredited facilities, which are the main data sources in this study. The strength or reliability of the present study lies in the wide perspective and the large sample of the data collected from all public and private (medical scheme) hospitals and facilities in Gauteng province. This discloses a broader scope of understanding VMMC coverage in Gauteng during COVID-19 and the importance thereof in combating HIV and/or AIDS.

## Conclusion

In 2020 during the COVID-19 pandemic, VMMC substantially declined in Gauteng province. However, Tshwane, one of the five districts in Gauteng, experienced an increase in VMMC, as a lesson to be learned about possible health systems resistance during crises. More research is needed to investigate the mechanism of resistance and the leadership role of the Tshwane district in sustaining VMMC during COVID-19, to benefit the entire health system. Also, studies need to assess the recovery of VMMC in Gauteng province post-COVID-19. Although the current study cannot fully ascertain that the disruption in VMMC in Gauteng province in 2020 was solely because of COVID-19, the pandemic remains a significant potential driver of the disruption observed in VMMC. Enhancing the prevention of HIV transmission is crucial and warrants a return of VMMC to its pre-pandemic level, as well as ensuring strategic planning to enable the continuum of VMMC during future emergencies.
